# Apple E3 ligase MdPUB23 mediates ubiquitin-dependent degradation of MdABI5 to delay ABA-triggered leaf senescence

**DOI:** 10.1093/hr/uhae029

**Published:** 2024-01-30

**Authors:** Fei Yang, Ling-Ling Zhao, Lai-Qing Song, Yuepeng Han, Chun-Xiang You, Jian-Ping An

**Affiliations:** Apple Technology Innovation Center of Shandong Province, College of Horticulture Science and Engineering, Shandong Agricultural University, Tai-An, 271018, Shandong, China; Yantai Academy of Agricultural Sciences, Yan-Tai 265599, Shandong, China; Yantai Academy of Agricultural Sciences, Yan-Tai 265599, Shandong, China; CAS Key Laboratory of Plant Germplasm Enhancement and Specialty Agriculture, Wuhan Botanical Garden, Hubei Hongshan Laboratory, The Innovative Academy of Seed Design of Chinese Academy of Sciences, Wuhan 430074, China; Apple Technology Innovation Center of Shandong Province, College of Horticulture Science and Engineering, Shandong Agricultural University, Tai-An, 271018, Shandong, China; Apple Technology Innovation Center of Shandong Province, College of Horticulture Science and Engineering, Shandong Agricultural University, Tai-An, 271018, Shandong, China; CAS Key Laboratory of Plant Germplasm Enhancement and Specialty Agriculture, Wuhan Botanical Garden, Hubei Hongshan Laboratory, The Innovative Academy of Seed Design of Chinese Academy of Sciences, Wuhan 430074, China

## Abstract

ABSCISIC ACID-INSENSITIVE5 (ABI5) is a core regulatory factor that mediates the ABA signaling response and leaf senescence. However, the molecular mechanism underlying the synergistic regulation of leaf senescence by ABI5 with interacting partners and the homeostasis of ABI5 in the ABA signaling response remain to be further investigated. In this study, we found that the accelerated effect of MdABI5 on leaf senescence is partly dependent on MdbHLH93, an activator of leaf senescence in apple. MdABI5 directly interacted with MdbHLH93 and improved the transcriptional activation of the senescence-associated gene *MdSAG18* by MdbHLH93. MdPUB23, a U-box E3 ubiquitin ligase, physically interacted with MdABI5 and delayed ABA-triggered leaf senescence. Genetic and biochemical analyses suggest that MdPUB23 inhibited MdABI5-promoted leaf premature senescence by targeting MdABI5 for ubiquitin-dependent degradation. In conclusion, our results verify that MdABI5 accelerates leaf senescence through the MdABI5-MdbHLH93-*MdSAG18* regulatory module, and MdPUB23 is responsible for the dynamic regulation of ABA-triggered leaf senescence by modulating the homeostasis of MdABI5.

## Introduction

Plants have evolved precise and complex regulatory mechanisms to cope with unfavorable conditions. The phytohormone abscisic acid (ABA) is considered an ‘anti-stress hormone’. When plants are exposed to abiotic stresses such as drought, salinity, and extreme temperature, the endogenous ABA content increases rapidly, which promotes seed dormancy, inhibits plant growth, and induces the expression of stress response-related genes, which helps plants cope with these stresses [1–3]. In addition to its role in the abiotic stress response, ABA plays an important role in regulating seed germination and dormancy, seedling establishment, stomatal movement, root growth, plant immunity, leaf senescence, fruit coloring, and fruit ripening [4–6].

The key components of the ABA signal transduction network have been preliminarily elucidated. ABA INSENSITIVE 1–5 (ABI1–ABI5) are key regulatory factors of the ABA signaling response. Cloning analysis of *ABI1–ABI5* in *Arabidopsis* has revealed that the *ABI1* and *ABI2* genes encode protein phosphatase 2Cs (PP2Cs), whereas ABI3, ABI4, and ABI5 proteins belong to the APETALA2 (AP2)/ETHYLENE RESPONSE FACTOR (ERF), and basic leucine zipper (bZIP) transcription factor (TF) families, respectively [7, 8]. The PYRABACTIN RESISTANCE 1 (PYR1)/PYR1-LIKE (PYL)/REGULATORY COMPONENTS OF ABA RECEPTORS (RCARs) have been identified as ABA receptors that belong to two independent groups [9, 10]. The plant-specific protein kinase SNF1-related protein kinase 2s (SnRK2s) are core components of the ABA receptor coupling core signaling pathway [11, 12]. The dual inhibition system consisting of PYR1/PYL/RCAR, PP2Cs, SnRK2s, and the substrates of SnRK2s constitutes the central transduction pathway of the ABA signaling response [13–15]. Under non-stress conditions, PP2Cs directly interact with and dephosphorylate SnRK2s, inhibiting SnRK2 kinase activity, preventing SnRK2s from activating their substrates, and thereby blocking the ABA signaling pathway and stress responses. When PYR1/PYL/RCAR sense ABA, they bind to PP2Cs to form complexes that inhibit the phosphatase activity of PP2Cs and dissociate the PP2Cs-SnRK2s complex. Activated SnRK2s activate the ABA signaling pathway and stress response processes by phosphorylating substrate proteins such as ABI5 or ABA-responsive element binding factors (ABFs) [16–18]. The transition of SnRK2s from the inactive dephosphorylated state to the active state is a key link in plant ABA signal transduction and stress responses.

Protein post-translational modifications such as ubiquitination, phosphorylation/dephosphorylation, and small ubiquitin-related modifier (SUMO)ylation play a critical role in the regulation of ABA signal transduction pathways [19–21]. Currently, interest in ubiquitination in the ABA signaling response is growing [22]. Ubiquitination involves a series of continuous catalytic reactions carried out by E1 ubiquitin-activating enzyme, E2 ubiquitin-conjugating enzyme, and E3 ubiquitin ligase [23, 24]. Because E3 ubiquitin ligase determines the specificity of target proteins, E3 has been studied more intensively. E3 ubiquitin ligases can be divided into three major groups according to their conserved domains: Really Interesting New Gene (RING), homologous to the E6AP carboxyl terminus (HECT), and Cullin-RING (CRL) [25, 26]. Ubiquitination mainly affects protein function, localization, and stability. Most studies of ubiquitination in the ABA signal transduction pathway have focused on the selective degradation of proteins. For example, the E3 ubiquitin ligases ABI3-interacting protein (AIP2) and KEEP ON GOING (KEG) repress ABA signaling by promoting the ubiquitin-dependent degradation of ABI4 and ABF1/3, respectively [27, 28]. RING FINGER OF SEED LONGEVITY1 (RSL1) and Plant U-box-type E3 ligase22/23 (PUB22/23) directly mediate ABA signaling through ubiquitination of the ABA receptors PYL4, PYR1, and PYL9, respectively [29, 30]. ABA promotes the turnover of phosphatase PP2CA by the ubiquitin ligase RING Domain Ligase1/5 (RGLG1/5) [31], and ABA inhibits ubiquitin-dependent degradation of MYB30, MYB96, and ABI5 by MYB30-Interacting E3 ligase1 (MIEL1) [32–34].

ABI5 is a bZIP TF, and *Arabidopsis* mutants with a faulty ABI5 exhibit an ABA-insensitive phenotype [35]. ABI5 is a transcriptional regulator that initiates the ABA signaling response by identifying and binding to ABA-responsive elements of target gene promoters [36]. Numerous studies have reported that ABI5 is involved in the regulation of seed development, seedling growth, and stress responses in *Arabidopsis* [37, 38]. MaABI5-like modulates chilling-induced softening disorders in banana fruits [39]. In apple, overexpression of *MdABI5* promotes anthocyanin biosynthesis, accelerates leaf senescence, and represses nitrate transport [40–42]. Protein post-translational modifications play an essential role in modulating the protein activity and turnover of ABI5. In *Arabidopsis*, the protein kinases BRASSINOSTEROID-INSENSITIVE 2 (BIN2), SOS2-like protein kinase5 (PSK5), and calcineurin B-like interacting protein kinase26 (CIPK26) phosphorylate and activate ABI5 [43–45], and the phosphatases PROTEIN PHOSPHATASE6 (PP6) and PP2A dephosphorylate ABI5 [46, 47]. *Arabidopsis* ABI5 protein may be a phosphorylated target of apple mitogen-activated protein kinase1 (MdMPK1) [48]. The SUMO E3 ligase SIZ1 mediates the sumoylation of ABI5 [49]. In the ubiquitination-mediated protein stability of ABI5, ABI five binding protein (AFP), KEG, CONSTITUTIVELY PHOTOMORPHOGENIC9 (COP9), DWD HYPERSENSITIVE TO ABA1/2 (DWA1/2), ABA-HYPERSENSITIVE DCAF1 (ABD1), MIEL1, and PUB8 promote the ubiquitin-dependent degradation of ABI5 [34, 50–55], and XPO1-Interacting WD40 protein1 (XIW1) and COP1 protect ABI5 from proteasome degradation [56, 57].

Leaf senescence is the last stage of plant growth and development, and is mainly characterized by chlorophyll degradation [58, 59]. Leaf senescence is strictly supervised by hormone signals, and ABA signaling plays an irreplaceable role in modulating leaf senescence [60, 61]. Leaf senescence induces increases in ABA content, and exogenous ABA application accelerates leaf senescence [62, 63]. Leaf senescence is modulated by a series of ABA response factors [64, 65]. In apple, MdABI5 and the basic helix–loop–helix (bHLH) TF MdbHLH93 promote ABA-triggered leaf senescence [41, 66], but the regulatory relationship between the two remains unclear. Here, we found that the regulatory effects of MdABI5 on leaf senescence were partially dependent on MdbHLH93. Further investigations suggested that MdABI5 interacted with MdbHLH93 and enhanced the transcriptional activation of the senescence-associated gene *MdSAG18* by MdbHLH93, thereby promoting leaf senescence. The U-box E3 ubiquitin ligase MdPUB23 contributed to the ubiquitin-dependent degradation of MdABI5 and delayed ABA-triggered leaf senescence. In conclusion, we confirmed that MdABI5 accelerates leaf senescence through the MdABI5-MdbHLH93-*MdSAG18* regulatory module and undergoes the ubiquitination regulation of MdPUB23.

## Results

### MdABI5 interacts with MdbHLH93

**Figure 1:**
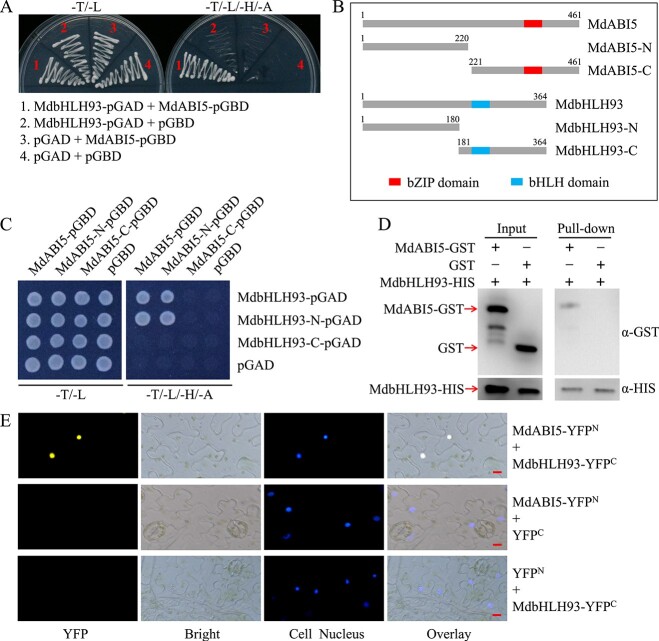
MdABI5 interacts with MdbHLH93. **A** Y2H analysis of interaction between MdABI5 and MdbHLH93. −T/−L, medium lacking Trp and Leu; −T/−L/-H/−A, medium lacking Trp, Leu, His, and Ade. **B** Schematic domain constructs of the MdABI5 and MdbHLH93 proteins. MdABI5, 1–461 aa; MdABI5-N, 1–220 aa; MdABI5-C, 221–461 aa; MdbHLH93, 1–364 aa; MdbHLH93-N, 1–180 aa; MdbHLH93-C, 181–364 aa. **C** Y2H analysis of interaction between truncated MdABI5 and truncated MdbHLH93. **D** Pull-down assays. **E** BiFC assays. All scale bars are 10 μm.

The MdABI5 TF is a key positive regulator of ABA-triggered leaf senescence [40] (Supplementary Data Fig. 1). To further explore the regulatory network of ABA-triggered leaf senescence, we employed the yeast screening system to search for MdABI5-interacting proteins associated with leaf senescence. The full-length coding sequence of *MdABI5* was assembled into the pGBT9 vector (MdABI5-pGBD) and used as the bait vector (Supplementary Data Table 1). Sequencing of positive colonies revealed that MdbHLH93, a bHLH TF that has been previously reported to accelerate leaf senescence [66], is a candidate interaction partner for MdABI5. To confirm the interaction between MdABI5 and MdbHLH93, the prey vector (MdbHLH93-pGAD) was generated by inserting the full-length coding sequence of *MdbHLH93* into the pGAD424 vector. The MdABI5-pGBD and MdbHLH93-pGAD recombinant plasmids were simultaneously transformed into yeast cells. Yeast two-hybrid (Y2H) assay results indicated that only yeast cells carrying both MdABI5-pGBD and MdbHLH93-pGAD were able to grow normally on selective medium, and lack of either led to the failure of yeast growth (Fig. 1A), suggesting that MdABI5 interacts with MdbHLH93 in yeast cells. Additional Y2H results revealed that the N-terminal fragments of both MdABI5 and MdbHLH93 are critical for their interaction (Fig. 1B and C).

Subsequently, pull-down assays were conducted to further corroborate the physical interaction between MdABI5 and MdbHLH93. *MdABI5* was fused to the pGEX4T-1 vector and *MdbHLH93* was ligated to the pET32a vector. The MdABI5-GST and MdbHLH93-HIS fusion proteins were generated through isopropyl-β-d-thiogalactoside (IPTG) induction. The corresponding fusion protein combinations were incubated in nickel affinity chromatography. Western blot results suggested that MdbHLH93 had affinity for MdABI5-GST protein, but did not bind to the GST control (Fig. 1D), indicating that MdABI5 physically interacts with MdbHLH93.

To further provide evidence for the interaction between MdABI5 and MdbHLH93, bimolecular fluorescence complementation (BiFC) assays *in planta* were conducted. MdABI5 and MdbHLH93 were cloned into the N-terminal and C-terminal YFP fragment, respectively, to generate MdABI5-YFP^N^ and MdbHLH93-YFP^C^. The recombinant plasmids were infiltrated into tobacco leaves through *Agrobacterium*-mediated transformation. A strong fluorescence signal was detected in the nuclei of tobacco leaf cells when MdABI5-YFP^N^ and MdbHLH93-YFP^C^ were expressed simultaneously (Fig. 1E), demonstrating that MdABI5 interacts with MdbHLH93 in the nucleus. Overall, these data demonstrate that MdbHLH93 is an interacting partner of MdABI5.

### *MdABI5* accelerates leaf senescence in a partially *MdbHLH93*-dependent manner

**Figure 2:**
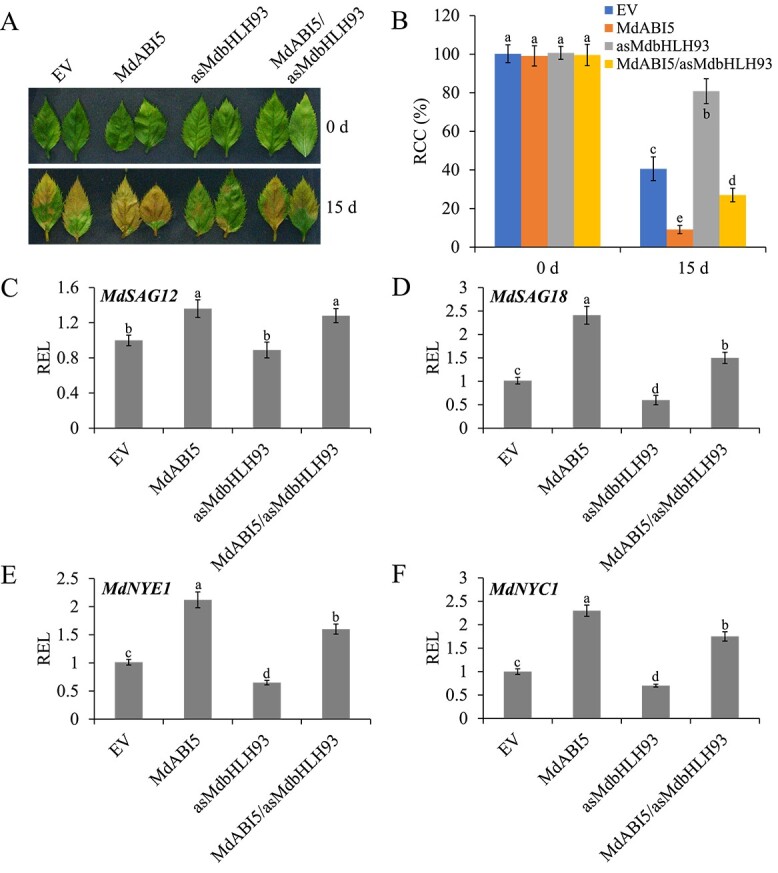
Acceleration of leaf senescence by MdABI5 is partly dependent on MdbHLH93. **A** Senescence phenotypes of *MdABI5-* and *MdbHLH93-*transgenic apple leaves before and after 15 days of dark treatment. Each treatment was performed in triplicate and each replicate comprised 8–10 apple leaves. Representative pictures are shown. EV, empty vector; MdABI5, *MdABI5*-overexpressing apple leaves; asMdbHLH93, apple leaves with expression of *MdbHLH93* suppressed; MdABI5/asMdbHLH93, leaves with *MdABI5* overexpressed and expression of *MdbHLH93* suppressed. **B** Relative chlorophyll content (RCC) in the apple leaves shown in **A**. The value for EV before dark treatment was set to 100% and used as the reference. **C**–**F** qRT–PCR analysis of the relative expression levels (REL) of *MdSAG12*, *MdSAG18*, *MdNYE1*, and *MdNYC1* in transgenic apple leaves. The value for EV was set to 1 and used as the reference. Three biological replicates were carried out with three technical replicates. Error bars denote standard deviations. Different lowercase letters indicate significant differences at *P* < 0.05 based on one-way ANOVA.

Given that MdbHLH93 interacts with MdABI5, we asked whether the regulation of leaf senescence by *MdABI5* depends on *MdbHLH93*. To test this, the *MdbHLH93* suppression vector was constructed and further expressed in *MdABI5*-overexpressing apple leaves (Supplementary Data Fig. 2A). Consistent with the results of previous studies [41, 66], overexpressing *MdABI5* accelerated the loss of chlorophyll in apple leaves, and the inhibition of *MdbHLH93* reduced chlorophyll loss in apple leaves compared with the empty vector controls (Fig. 2A and B; Supplementary Data Fig. 3). The suppression of *MdbHLH93* expression could alleviate the promoting effect of *MdABI5* on chlorophyll degradation in apple leaves (Fig. 2A and B), suggesting that MdbHLH93 plays an important role in MdABI5-mediated leaf senescence. Transcriptional analysis of senescence-associated genes showed that inhibition of the expression of *MdbHLH93* decreased the transcriptional activation of *MdSAG18*, *MdNYE1*, and *MdNYC1* by MdABI5 (Fig. 2C–F; Supplementary Data Fig. 4). The above results reveal that the regulation of leaf senescence by MdABI5 is partly dependent on MdbHLH93.

### MdABI5 promotes DNA-binding activity of MdbHLH93 to *MdSAG18*

**Figure 3:**
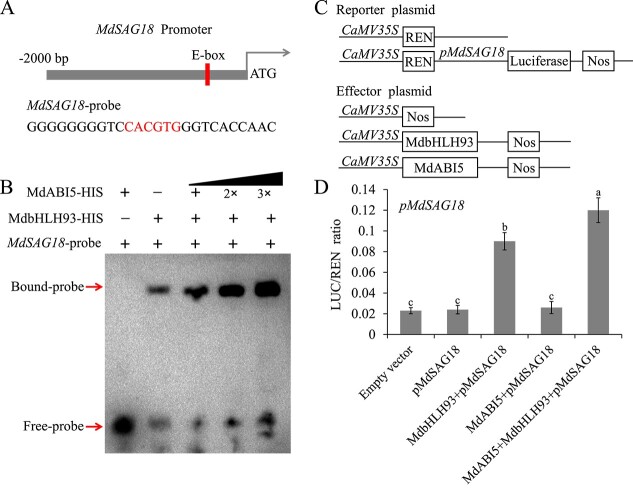
MdABI5 affects the transcriptional function of MdbHLH93. **A** Schematic diagram of the *MdSAG18* promoter. **B** EMSAs showing that MdABI5 enhanced the binding of MdbHLH93 to the *MdSAG18* promoter. **C** Schematic diagram of the reporters and effectors. **D** Dual luciferase assays. The LUC/REN ratio for the empty vector was used as the reference. Error bars denote standard deviations. Different lowercase letters indicate significant difference at *P* < 0.05 based on one-way ANOVA.

As MdABI5 interacts with MdbHLH93 and MdABI5 promotes leaf senescence in a manner partially dependent on MdbHLH93, we investigated whether MdABI5 affects the transcriptional function of MdbHLH93. *MdSAG18* is the target gene of MdbHLH93 [66]. Next, we determined the effect of MdABI5 on the transcriptional activation of *MdSAG18* by MdbHLH93 using electrophoretic mobility shift assays (EMSAs). We noted that MdbHLH93 bound to the *MdSAG18* promoter, whereas MdABI5 did not (Fig. 3A and B; Supplementary Data Table 2). Notably, gradient addition of MdABI5 gradually enhanced the binding strength of MdbHLH93 to the *MdSAG18* promoter (Fig. 3B; Supplementary Data Fig. 5). These results indicate that MdABI5 promotes the DNA-binding activity of MdbHLH93 to the *MdSAG18* promoter *in vitro*.

To further elucidate the synergistic effects of MdABI5 on MdbHLH93, dual luciferase assays were performed in tobacco leaves. The promoter sequence of *MdSAG18* was fused to the pGreen0800-LUC vector to generate the reporter plasmid. The full-length coding sequences of *MdbHLH93* and *MdABI5* were individually embedded into the pGreen62-SK vector to generate the effector plasmids (Fig. 3C). As expected, MdbHLH93 activated the expression of the *MdSAG18* promoter, and MdABI5 appeared to have no significant effect on the expression of the *MdSAG18* promoter (Fig. 3D). Co-expression of MdABI5 with MdbHLH93 further enhanced the expression of the *MdSAG18* promoter compared with the expression of MdbHLH93 alone (Fig. 3D). These findings suggest that MdABI5 facilitates the transcriptional activity of MdbHLH93 on *MdSAG18*. However, MdbHLH93 did not appear to have a significant effect on MdABI5 transcriptional activation of *MdNYE1* and *MdNYC1* (Supplementary Data Figs 6 and 7).

### MdPUB23 interacts with MdABI5

**Figure 4:**
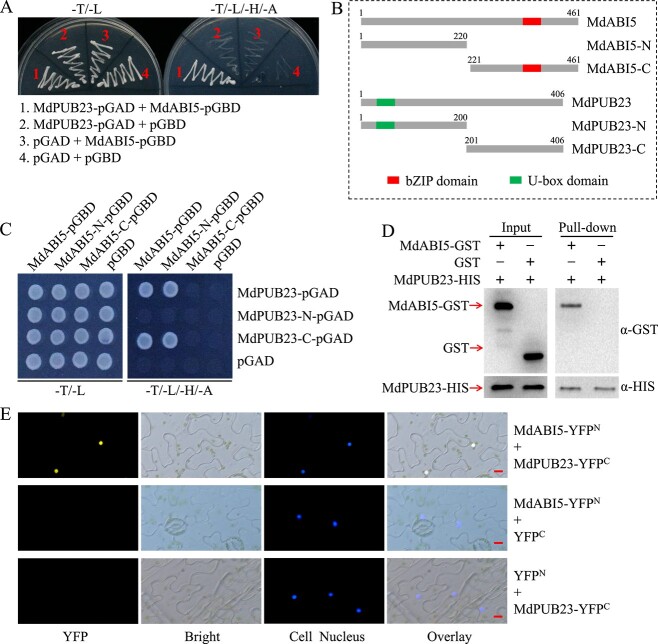
MdPUB23 interacts with MdABI5. **A** Y2H analysis of interaction between MdPUB23 and MdABI5. −T/−L, medium lacking Trp and Leu; −T/−L/−H/−A, medium lacking Trp, Leu, His, and Ade. **B** Schematic domain constructs of the MdABI5 and MdPUB23 proteins. MdABI5, 1–461 aa; MdABI5-N, 1–220 aa; MdABI5-C, 221–461 aa; MdPUB23, 1–406 aa; MdPUB23-N, 1–200 aa; MdPUB23-C, 201–406 aa. **C** Y2H analysis of the interaction between truncated MdPUB23 and truncated MdABI5. **D** Pull-down assays. **E** BiFC assays. All scale bars are 10 μm.

ABA treatment not only induces *MdABI5* expression at the transcriptional level, but also improves the protein stability of MdABI5 (Supplementary Data Fig. 8). To further investigate the role of MdABI5 in ABA signaling, we screened for E3 ubiquitin ligases that may be involved in regulating the stability of MdABI5 using a yeast system. The U-box-type E3 ubiquitin ligase MdPUB23 was identified [67] (Supplementary Data Table 1). MdPUB23-pGAD and MdABI5-pGBD recombinant plasmids were constructed, and the interaction between MdPUB23 and MdABI5 was confirmed by Y2H assays. The results showed that the C-terminal fragment of MdPUB23 interacts with the N-terminal fragment of MdABI5 in yeast cells (Fig. 4A–C).

To further verify the MdPUB23–MdABI5 interaction, pull-down and BiFC assays were performed. For the pull-down assays, the MdABI5-GST and MdPUB23-HIS fusion proteins were incubated in nickel affinity chromatography. The results revealed that MdPUB23 could chelate the MdABI5 protein (Fig. 4D). For the BiFC assays, YFP fluorescence signal was observed in the nuclei of tobacco leaf cells when MdABI5-YFP^N^ was co-expressed with MdPUB23-YFP^C^ (Fig. 4E; Supplementary Data Fig. 9). The above results reveal that MdPUB23 physically interacts with MdABI5.

### MdPUB23 delays abscisic acid-induced leaf senescence

**Figure 5:**
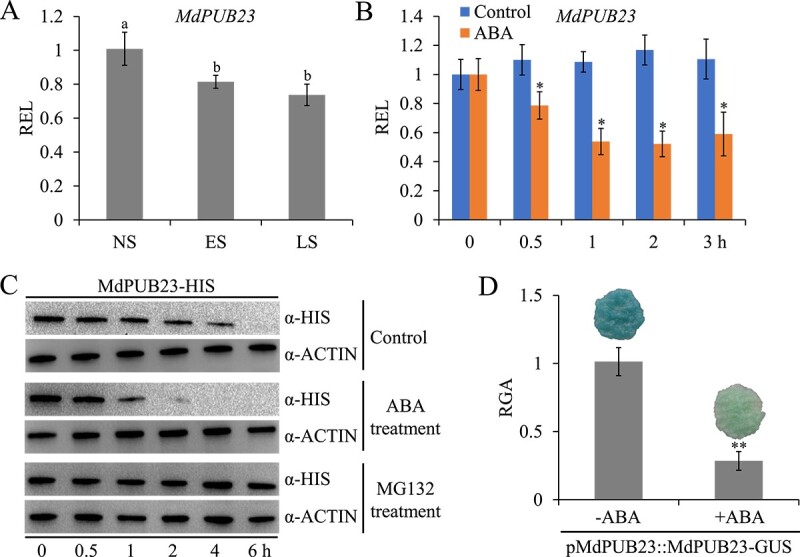
Expression pattern of MdPUB23 during leaf senescence and after ABA treatment. **A** qRT–PCR analysis of the relative expression level (REL) of *MdPUB23* during leaf senescence. NS, non-senescing, ES, early-senescing, LS, late-senescing. The value for NS was set to 1 and used as the reference. Three biological replicates were carried out with three technical replicates. **B** qRT–PCR analysis of the REL of *MdPUB23* after 50 μM ABA treatment for 12 h. The value for the control without any treatment was set to 1 and used as the reference. Three biological replicates were carried out with three technical replicates. **C** Western blot analysis of degradation of MdPUB23-HIS protein after treatment with 50 μM ABA or MG132 for 6 h. **D** GUS staining and relative GUS activity (RGA) of *MdPUB23*-transgenic apple calli in the absence and presence of ABA. The GUS activity for the apple calli without ABA treatment was set to 1 and used as the reference. Error bars denote standard deviations. Different lowercase letters indicate significant differences at *P* < 0.05 based on one-way ANOVA. ^*^P < 0.05, ^**^P < 0.01; *t*-test.

The leaves of 40-day-old apple tissue culture seedlings at different senescence stages were collected and named as non-senescing (NS), early-senescing (ES), and late-senescing (LS) [66, 68, 69]. We detected that the transcription level of *MdPUB23* decreased during leaf senescence (Fig. 5A). ABA exposure reduced the expression of *MdPUB23* and the stability of MdPUB23 protein (Fig. 5B–D). These results indicate that *MdPUB23* may play a role in regulating leaf senescence and the ABA response.

**Figure 6:**
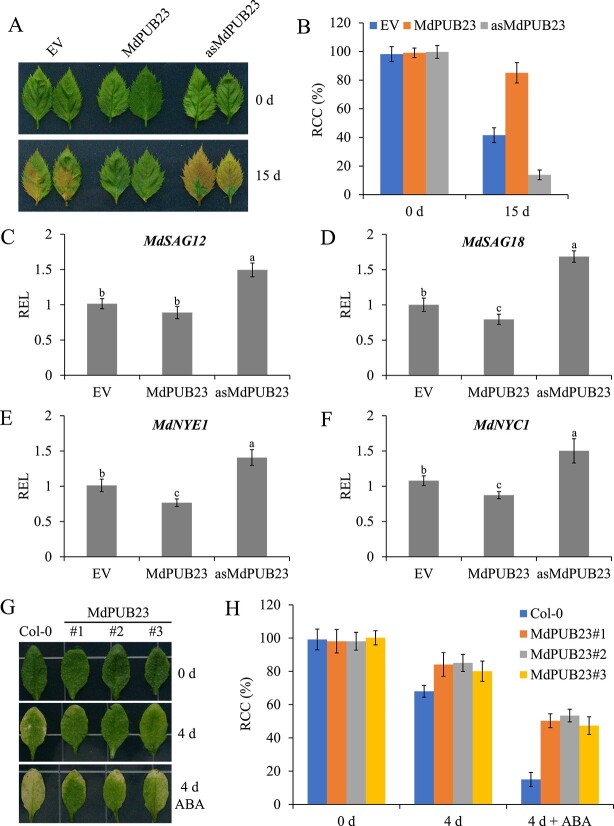
*MdPUB23* negatively regulates leaf senescence. **A** Senescence phenotypes of *MdPUB23*-transgenic apple leaves before and after 15 days of dark treatment. Each treatment was performed in triplicate and each replicate comprised 8–10 apple leaves. Representative pictures are shown. EV, empty vector; MdPUB23, *MdPUB23*-overexpressing apple leaves; asMdPUB23, apple leaves with the expression of *MdPUB23* suppressed. **B** Relative chlorophyll content (RCC) in the apple leaves shown in **A**. The value for EV before dark treatment was set to 100% and used as the reference. **C**–**F** qRT–PCR analysis of relative expression levels (REL) of *MdSAG12*, *MdSAG18*, *MdNYE1*, and *MdNYC1* in transgenic apple leaves. The value for EV was set to 1 and used as the reference. Three biological replicates were carried out with three technical replicates. **G** Senescence phenotypes of *MdPUB23*-transgenic *Arabidopsis* leaves with or without 30 μM ABA treatment under dark conditions for 4 days. Each treatment was performed in triplicate and each replicate comprised 8–10 *Arabidopsis* leaves. Representative pictures are shown. Col-0, wild type; MdPUB23#1, #2, and #3, *MdPUB23*-overexpressing *Arabidopsis* leaves. **H** RCC in the *Arabidopsis* leaves shown in **G**. The value for Col-0 at 0 days was set to 100% and used as the reference. Error bars denote standard deviations. Different lowercase letters indicate significant differences at *P* < 0.05 based on one-way ANOVA.

To explore the regulatory role of *MdPUB23* in leaf senescence, we constructed *MdPUB23* overexpression and suppression vectors and transient transformation was performed in apple leaves (Supplementary Data Fig. 2B). Senescence assays of apple leaves showed that overexpression of *MdPUB23* suppressed leaf senescence by downregulating the expression of the senescence-associated genes *MdSAG18*, *MdNYE1*, and *MdNYC1*, whereas the opposite phenotypes were observed when the expression of *MdPUB23* was inhibited (Fig. 6A–F). Transcriptional analysis of senescence-associated genes in *MdPUB23*-transgenic apple calli revealed that MdPUB23 negatively regulated the expression of *MdSAG18*, *MdNYE1*, and *MdNYC1* (Supplementary Data Fig. 10). Transgenic *Arabidopsis* seedlings overexpressing *MdPUB23* were generated and employed for leaf senescence assays (Supplementary Data Fig. 2C). The senescence assays of *Arabidopsis* detached leaves revealed that overexpression of *MdPUB23* improved the stay-green phenotypes (Fig. 6G and H).

To further verify the regulatory role of *MdPUB23* in ABA-triggered leaf senescence, wild-type (WT) and *MdPUB23*-transgenic *Arabidopsis* leaves were treated with exogenous ABA. The results of senescence assays showed that ABA treatment reduced the chlorophyll content of WT *Arabidopsis* from 65 to 15%, and the chlorophyll content of *MdPUB23*-transgenic *Arabidopsis* decreased from 80 to 50% (Fig. 6G and H), indicating that *MdPUB23* plays a negative regulatory role in ABA-triggered leaf senescence.

### MdPUB23 promotes the ubiquitin-dependent degradation of MdABI5

**Figure 7:**
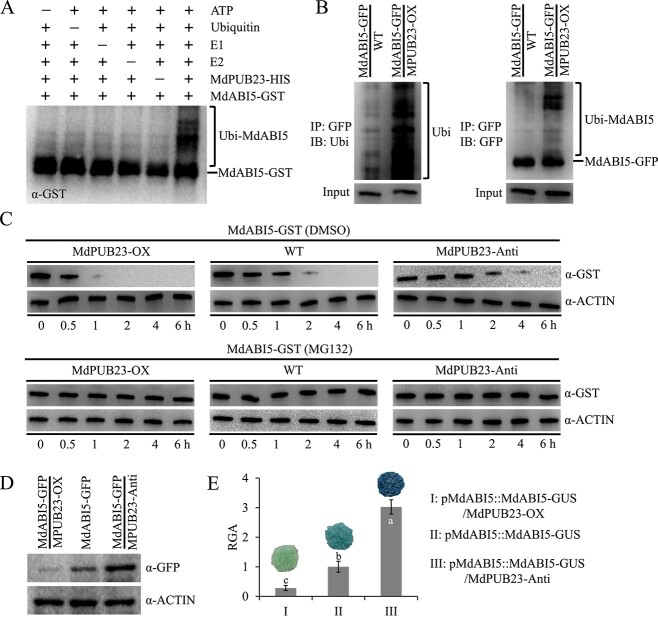
MdPUB23 ubiquitinates MdABI5 and facilitates its degradation. **A***In vitro* ubiquitination assays. **B** Ubiquitination detection *in vivo*. **C** Protein degradation assays *in vitro*. **D** Protein abundance of MdABI5 in MdABI5-GFP, MdABI5-GFP/MdPUB23-OX, and MdABI5-OX/MdPUB23-Anti transgenic apple calli. **E** GUS staining and relative GUS activity (RGA) of *MdABI5* single-transgenic calli and *MdABI5*-*MdPUB23* co-transgenic calli. GUS activity of the *MdABI5* single-transgenic calli was set to 1 and used as the reference. Error bars denote standard deviations. Different lowercase letters indicate significant differences at *P* < 0.05 based on one-way ANOVA.

Given that MdPUB23 encodes a U-box E3 ubiquitin ligase, we next investigated whether MdPUB23 mediates the ubiquitination regulation of MdbHLH93. Ubiquitination assays *in vitro* showed that in the presence of ATP, ubiquitin, E1, E2, and MdPUB23-HIS, the MdABI5 fusion protein had a ubiquitination tail (Fig. 7A), indicating that MdABI5 is the ubiquitination substrate of MdPUB23 *in vitro*. In addition, the MdABI5-GFP protein was precipitated from MdABI5-GFP and MdABI5-GFP/MdPUB23-OX transgenic apple calli and the ubiquitination of MdABI5 was detected using anti-Ubi and anti-GFP antibodies (Supplementary Data Fig. 2D). As shown in Fig. 7B, the ubiquitination level of MdABI5 was markedly higher in MdABI5-GFP/MdPUB23-OX co-transgenic calli than in MdABI5-GFP single-transgenic calli (Fig. 7B), indicating that MdPUB23 promotes the ubiquitination of MdABI5 *in vivo*.

We then analyzed the effect of MdPUB23 on MdABI5 protein turnover. The MdABI5-GST fusion protein was mixed and incubated with total proteins extracted from WT, MdPUB23-OX, and MdPUB23-Anti apple calli. Western blot analysis showed that overexpressing *MdPUB23* accelerated the ubiquitin-dependent degradation of MdABI5, and the degradation rate of MdABI5 was repressed in MdPUB23-Anti calli (Fig. 7C; Supplementary Data Fig. 11). The results of the protein abundance test *in vivo* showed that overexpression of *MdPUB23* could decrease the protein level of MdABI5, and inhibition of *MdPUB23* could increase the abundance of MdABI5 protein (Fig. 7D and E). These results indicate that MdPUB23 promotes the ubiquitin-dependent degradation of MdABI5.

### MdPUB23 antagonizes the function of MdABI5

**Figure 8:**
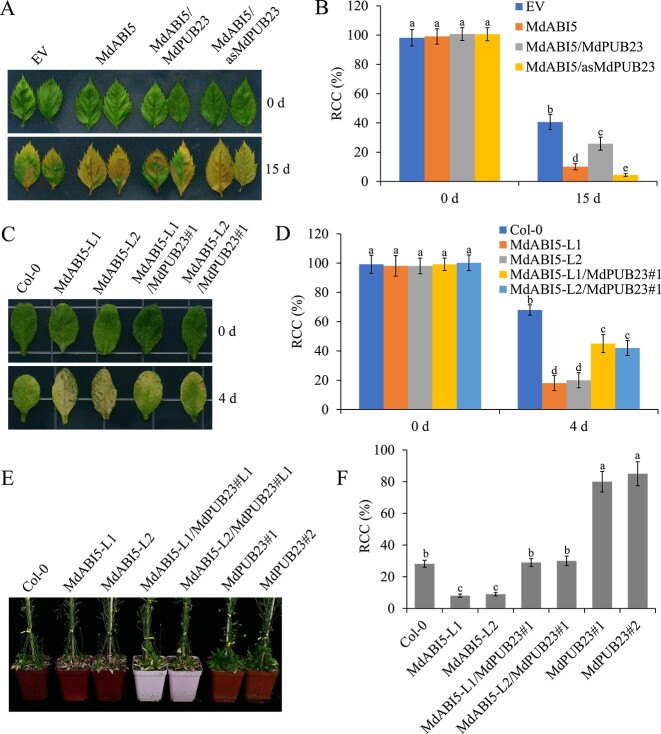
*MdPUB23* antagonizes *MdABI5*-mediated leaf senescence. **A** Senescence phenotypes of *MdPUB23*- and *MdABI5*-transgenic apple leaves before and after 15 days of dark treatment. Each treatment was performed in triplicate and each replicate comprised 8–10 apple leaves. Representative pictures are shown. EV, empty vector; MdABI5, *MdABI5*-overexpressing apple leaves; MdABI5/MdPUB23, leaves with both *MdABI5* and *MdPUB23* overexpressed; MdABI5/asMdPUB23, leaves with *MdABI5* overexpressed and the expression of *MdPUB23* suppressed. **B** Relative chlorophyll content (RCC) in the apple leaves shown in **A**. The value for EV before dark treatment was set to 100% and used as the reference. **C** Senescence phenotypes of *MdPUB23* and *MdABI5* transgenic *Arabidopsis* leaves before and after 4 days of dark treatment. Col-0, wild type; MdABI5-L1 and L2, *MdABI5*-overexpressing *Arabidopsis* leaves; MdABI5-L1/MdPUB23#1 and MdABI5-L2/MdPUB23#1, *MdABI5* and *MdPUB23* co-expression in *Arabidopsis* leaves. **D** RCC in the *Arabidopsis* leaves shown in **C**. The value for Col-0 at 0 days was set to 100% and used as the reference. **E** Senescence phenotypes of 7-week-old Col-0 and transgenic *Arabidopsis* seedlings grown under long-day conditions. Each treatment was performed in triplicate and each replicate comprised six to eight *Arabidopsis* seedlings. Representative pictures are shown. Col-0, wild type; MdABI5-L1 and L2, *MdABI5*-overexpressing *Arabidopsis* seedlings; MdABI5-L1/MdPUB23#1 and MdABI5-L2/MdPUB23#1, *MdABI5* and *MdPUB23* co-expression in *Arabidopsis* seedlings; MdPUB23#1 and #2, *MdPUB23*-overexpressing *Arabidopsis* seedlings. **F** RCC in the *Arabidopsis* leaves shown in **E**. Error bars denote standard deviations. Different lowercase letters indicate significant differences at *P* < 0.05 based on one-way ANOVA.

Based on the antagonistic roles of *MdPUB23* and *MdABI5* in leaf senescence regulation and the MdPUB23-mediated ubiquitin-dependent degradation of MdABI5, we asked whether *MdPUB23* affects the regulation of leaf senescence by *MdABI5*. To test this, we obtained transient transgenic apple leaves co-expressing *MdABI5* and *MdPUB23* to perform leaf senescence assays. Overexpression of *MdPUB23* delayed *MdABI5*-induced leaf senescence, whereas inhibition of *MdPUB23* accelerated leaf senescence promoted by *MdABI5* (Fig. 8A and B). In addition, transgenic *Arabidopsis* seedlings with co-expression of *MdABI5* and *MdPUB23* were generated to conduct detached leaf senescence assays (Supplementary Data Fig. 2E). As expected, overexpression of *MdPUB23* attenuated the promoting effect of *MdABI5* on leaf senescence (Fig. 8C and D). We also observed the leaf senescence of *MdABI5* and *MdPUB23*-transgenic *Arabidopsis* seedlings under normal growth conditions. *MdABI5* accelerated leaf senescence and *MdPUB23* delayed leaf senescence; the co-expression of *MdPUB23* and *MdABI5* reduced the promoting effect of *MdABI5* on leaf senescence (Fig. 8E and F). The above results suggest that *MdPUB23* antagonizes the promoting effect of *MdABI5* on leaf senescence by intensifying the ubiquitin-dependent degradation of MdABI5.

## Discussion

Leaf senescence is the final stage of leaf growth and an irreversible development process; it thus must be strictly regulated. When the leaves are aged, the energy conversion efficiency of the leaves is greatly reduced, and the effective nutrients in the aged leaves return to the young organs in time for their secondary distribution [58, 63]. Therefore, study of the mechanism underlying the regulation of leaf senescence is important for improving crop yield. Numerous studies have reported that ABA, a stress hormone, plays a positive role in regulating leaf senescence [70]. Understanding the mechanism of leaf senescence mediated by ABA is essential for clarifying the regulatory effects of abiotic stresses on leaf growth. At present, the regulation of leaf senescence by ABA is the subject of extensive research. Overexpression of the ABA receptor *PYL9* in *Arabidopsis* causes premature leaf senescence [62]. NAC and bZIP TFs play key regulatory roles in ABA-induced leaf senescence by mediating ABA biosynthesis or the expression of senescence-associated genes [41, 71–75]. In *Arabidopsis* and apple, ABI5 acts as a core regulator of ABA signal transduction and promotes ABA-triggered leaf senescence by activating *NYE1* and *NYC1* expression [41, 76]. ABI5 can work in tandem with MED16/25, VQ18/26, INDUCER OF CBF EXPRESSION1 (ICE1), BRI1-EMS-SUPPRESSOR1 (BES1), ELONGATED HYPOCOTYL5 (HY5), and MYB30 in *Arabidopsis* [34, 38, 77–81]. In apple, several different types of TFs mediate the transcriptional activity of MdABI5 in ABA-mediated leaf senescence regulation. MdbZIP44, MdWRKY40, and MdBBX37 accelerate ABA-induced leaf senescence by combining with MdABI5 [41, 68]. MdBBX22 downregulates *MdABI5* expression by directly interfering with the transcriptional activity of MdABI5 and by inhibiting the transcriptional activation of *MdABI5* by MdHY5 [41]. However, the mechanism by which MdABI5 regulates leaf senescence remains unclear. MdbHLH93 has been identified as an activator of leaf senescence and is involved in ABA-triggered leaf senescence by stimulating the expression of *MdSAG18* [66]. In this study, inhibition of the expression of *MdbHLH93* attenuated the promoting effect of MdABI5 on leaf senescence (Fig. 2). Further investigations demonstrated that MdABI5 physically interacted with MdbHLH93 to promote the transcriptional activity of MdbHLH93 (Figs 1and 3). The results suggested that MdABI5-promoted leaf senescence can not only directly activate the expressions of *MdNYE1* and *MdNYC1* but can also mediate the expression of *MdSAG18* combined with MdbHLH93. We hypothesized that the MdABI5-MdbHLH93-*MdSAG18* and MdABI5-*MdNYE1*/*MdNYC1* modules might play equally important roles in leaf senescence triggered by ABA, which ensures a timely response to external stress.

**Figure 9:**
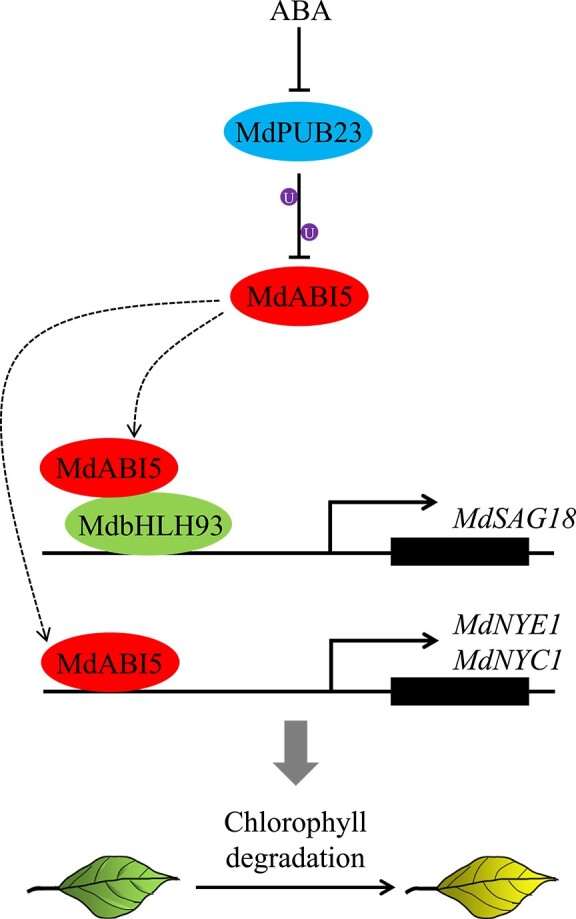
A model showing the role of *MdPUB23* in the regulation of leaf senescence. MdPUB23 delays leaf senescence by promoting turnover of MdABI5. In response to ABA, inhibition of MdPUB23 results in release of MdABI5. MdABI5 physically associates with MdbHLH93 to enhance its transcriptional activity on *MdSAG18*, thereby promoting leaf senescence. In addition, activated MdABI5 can also accelerate leaf senescence by directly activating the expression of *MdNYE1* and *MdNYC1*.

Plants respond to external stress by activating ABA signals to induce leaf senescence. However, when environmental conditions improve, plants must balance stress responses and growth by negatively regulating ABA signaling to ensure that signaling is not over-amplified or does not last too long. Therefore, study of the negative regulatory system of ABA signaling will enhance our understanding of ABA response pathways. ABA treatment not only induces *MdABI5* transcription but also promotes the accumulation of MdABI5 protein (Supplementary Data Fig. 8). ABA expression patterns of ABI5 in *Arabidopsis* and apple are similar [82], suggesting that ABI5 responses to ABA are conserved across species. *Arabidopsis* ABI5 protein turnover is regulated by the 26S-proteasome pathway, and the E3 ubiquitin ligases KEG, MIEL1, and PUB8 are involved in this process [34, 52, 55]. However, the role of MdABI5 in the regulation of leaf senescence at the post-translational level has not yet been clarified. In this study, the apple U-box E3 ligase MdPUB23 was found to directly interact with MdABI5 to delay ABA-triggered leaf senescence (Figs 4and 6). MdPUB23 could antagonize MdABI5-mediated leaf senescence by enhancing the ubiquitin-dependent degradation of MdABI5 (Figs 7and 8). Unlike the ABA-activated PUB8 regulation of ABI5 ubiquitination in *Arabidopsis* [55], MdPUB23 mediates MdABI5 degradation in the absence of ABA, and ABA inhibits MdPUB23 expression in apple (Fig. 5). This work pattern is similar to that of the MIEL1-ABI5 ubiquitination module [34]. In previous studies, MdPUB23 was shown to reduce cold stress resistance in apple by directly targeting MdICE1 [67]. The involvement of MdPUB23 in leaf senescence mediated by ABA has further enriched the regulatory function of MdPUB23. Which protein components are involved in the stability regulation of MdPUB23 protein remains to be further studied.

In conclusion, our data confirm that MdABI5 promotes leaf senescence through the MdABI5-MdbHLH93-*MdSAG18* module and undergoes the ubiquitination regulation of MdPUB23. A model was built to depict the involvement of MdABI5 in ABA-triggered leaf senescence (Fig. 9). MdABI5 not only accelerates leaf senescence by stimulating the expression of *MdNYE1* and *MdNYC1*, but also co-activates *MdSAG18* expression with MdbHLH93. MdPUB23 degrades MdABI5 by ubiquitination and inhibits leaf senescence. In the presence of ABA, ABA initiates leaf senescence under regulation by MdABI5 by inhibiting MdPUB23 expression and releasing MdABI5. Based on our previous studies showing that leaf senescence is accelerated by MdABI5 in collaboration with other interaction partners, we suggest that MdABI5 may play a central role in ABA-induced leaf senescence (Supplementary Data Fig. 12). Elucidating the regulatory network of leaf senescence mediated by MdABI5 will help us more comprehensively analyze the molecular mechanism of leaf senescence triggered by ABA, and provide a reference for regulating the leaf senescence cycle to improve crop yield in the future.

## Conclusion

MdABI5 promotes ABA-triggered leaf senescence through the MdABI5-MdbHLH93-MdSAG18 regulatory module. E3 ligase MdPUB23 mediates ubiquitin-dependent degradation of MdABI5 to delay ABA-induced leaf senescence. Our results not only reveal a new mechanism of leaf senescence induced by ABA, but also provide insights into the ubiquitination modification of ABI5.

## Materials and methods

### Plant materials

Apple tissue culture seedlings (*Malus* × *domestica*, GL-3) were grown under long-day conditions (16-h light/8-h dark) at 24°C and subcultured every 40 days. *Arabidopsis thaliana* Col-0 seedlings were grown under long-day conditions at 22°C.

### Plasmid construction and genetic transformation

The full-length coding sequences of *MdABI5* and *MdPUB23* were assembled into the pCXSN-GFP and pRI101 vectors, respectively, to construct overexpression plasmids. The designated sequences of *MdPUB23* and *MdbHLH93* were individually cloned into the pRI101 vector to generate suppression plasmids. Primers used for plasmid construction are shown in Supplementary Data Table 3.

For transient genetic transformation of apple leaves, leaves were incubated with *Agrobacterium* solution containing the designated plasmids and treated with a vacuum for 0.5 h. Transgenic *Arabidopsis* seedlings were obtained by dipping inflorescences in *Agrobacterium* solution containing the overexpression plasmid.

### Senescence assays

The detached leaves of 40-day-old apple seedlings and 3-week-old *Arabidopsis* seedlings were used for dark-induced senescence assays. The senescence degree of leaves was evaluated by measuring the chlorophyll content. For *Arabidopsis* leaf senescence induced by ABA, *Arabidopsis* leaves were sprayed with ABA solution in advance.

### Determination of chlorophyll content

Chlorophyll was extracted using a chlorophyll extract solution with 95% ethanol. Leaves or seedlings were cut up and soaked in a chlorophyll extract solution for 24 h. The absorbance of the chlorophyll solution at 649 and 665 nm was determined.

### Yeast two-hybrid, pull-down, and bimolecular fluorescence complementation assays

Y2H assays were performed using a Y2H Gold Yeast system (Clontech, CA, USA) [69, 83]. The transformed yeast cells were grown on a selective medium (SD/−T/−L) (Takara, Dalian, China), and yeast cells cultured for 3 days were transferred to SD/−T/−L/−H/−A medium (Takara) for another 3 days.

Pull-down assays were performed using a Pierce™ His Protein Interaction Pull-down Kit (ThermoFisher Scientific, Waltham, MA, USA) [69, 83]. The recombinant plasmids were transformed into *Escherichia coli* BL21 cells (TransGen Biotech, Beijing, China) and the fusion proteins were obtained by IPTG induction. The corresponding fusion protein combinations were incubated in nickel affinity chromatography. The eluent was tested with anti-GST and anti-HIS antibodies (Abmart, Shanghai, China).

For the BiFC assay, *Agrobacterium* solution containing recombinant plasmids was injected into tobacco leaves. The transformed tobacco plants were cultured under light conditions for 2 days. The fluorescence signals were observed using confocal microscopy.

### Identification of proteins interacting with MdABI5

The yeast library was prepared by Shanghai OE Biotech Co., Ltd using apple seedlings. For yeast library screening, the bait vector and yeast library were simultaneously transformed into yeast cells. The transformed yeast cells were grown on SD/−T/−L/−H/−A medium (Takara). Positive colonies were identified by PCR and analyzed by sequencing. Potential proteins that interact with MdABI5, including MdbHLH93, MdPUB23, MdbHLH3, MdBBX22, MdbZIP44, MdWRKY40, MdZAT10, and MdTCP46, can be found in Supplementary Data Table 1.

### Electrophoretic mobility shift and dual luciferase assays

EMSAs were carried out using an EMSA/Gel-Shift Kit (Beyotime) [69]. The fusion proteins were generated by IPTG induction. Biotin was tagged to DNA fragments using an EMSA Probe Biotin Labeling Kit (Beyotime, Shanghai, China) according to the manufacturer’s instructions. The fusion protein was incubated with the labeled probe at 24°C in the binding buffer for 30 min.

For the dual luciferase assay, effector and reporter plasmids were expressed in tobacco leaves by *Agrobacterium*-mediated genetic transformation. The LUC/REN ratio was determined using a Dual-Luciferase Reporter Assay System (Promega, Madison, WI, USA) according to the manufacturer’s instructions.

### Ubiquitination assays *in vitro* and protein degradation assays *in vitro*

ATP, ubiquitin, E1, and E2 were purchased from Sigma (Sigma–Aldrich, St Louis, MO, USA). For the *in vitro* ubiquitination assay, the above experimental samples were incubated at 30°C for 10 h.

For protein degradation assays *in vitro*, total proteins from WT and *MdPUB23*-transgenic apple calli were extracted with protein extract composed of 25 mM Tris–HCl, 10 mM NaCl, 10 mM MgCl_2_, 5 mM DTT, and 10 mM ATP. The MdABI5-GST fusion protein was incubated with total proteins at 30°C for 6 h. Degradation of MdABI5 protein was detected using anti-GST antibody (Abmart). For MG132 treatment, total proteins were pretreated with 50 μM MG132 for 0.5 h.

### Protein abundance determination and ubiquitination analysis *in vivo*

The MdABI5-GFP protein was extracted from MdABI5-GFP, MdABI5-GFP/MdPUB23-OX, and MdABI5-GFP/MdPUB23-Anti transgenic apple calli (15 days old/5 g). The protein abundance of MdABI5 was determined using anti-GFP antibody (Abmart).

For *in vivo* ubiquitination analysis, the MdABI5-GFP protein was precipitated from MdABI5-GFP and MdABI5-GFP/MdPUB23-OX transgenic apple calli using an Immunoprecipitation Kit (ThermoFisher Scientific). The ubiquitination level of MdABI5 was evaluated using anti-Ubi and anti-GFP antibodies (Abmart).

### GUS staining and activity determination

Apple calli carrying the recombinant plasmids were stained with a GUS Staining Kit (Solarbio, Beijing, China) according to the manufacturer’s instructions and GUS activity was determined with a fluorescence spectrophotometer (ThermoFisher Scientific).

## Acknowledgements

This work was financially supported by grants from the Natural Science Foundation of China (32372642), the Development Plan of the Youth Innovation Team of the Higher Education Institutions in Shandong Province (2022KJ326), and Wuhan Botanical Garden Scientific Research Support Project (E3559901).

## Author contributions

J.P.A. conceived and designed the experiments. F.Y., L.L.Z., and J.P.A. performed the research. C.X.Y., L.Q.S., Y.H., and J.P.A. analyzed the data. J.P.A. wrote the paper.

## Data availability

All the data generated or analyzed during this study are included in this published article. The apple gene sequences in this study can be obtained according to the following accession numbers: *MdbHLH93* (MDP0000644807), *MdABI5* (LOC103430245), *MdPUB23* (MDP0000773851), *MdSAG12* (MDP0000138228), *MdSAG18* (MDP0000274609), *MdNYE1* (MDP0000322543), and *MdNYC1* (MDP0000124013).

## Conflict of interest

The authors declare no competing interests.

## Supplementary Material

Web_Material_uhae029
