# Supplementary material for: Apple E3 ligase MdPUB23 mediates ubiquitin-dependent degradation of MdABI5 to delay ABA-triggered leaf senescence
Source: Hortic Res. 2024 Jan 30;11(4):uhae029. doi: 10.1093/hr/uhae029 (PMC10995623; doi:10.1093/hr/uhae029)
Supplement: Web_Material_uhae029 [file web_material_uhae029.zip › Supplemental information.docx]

**Supplemental Figures**


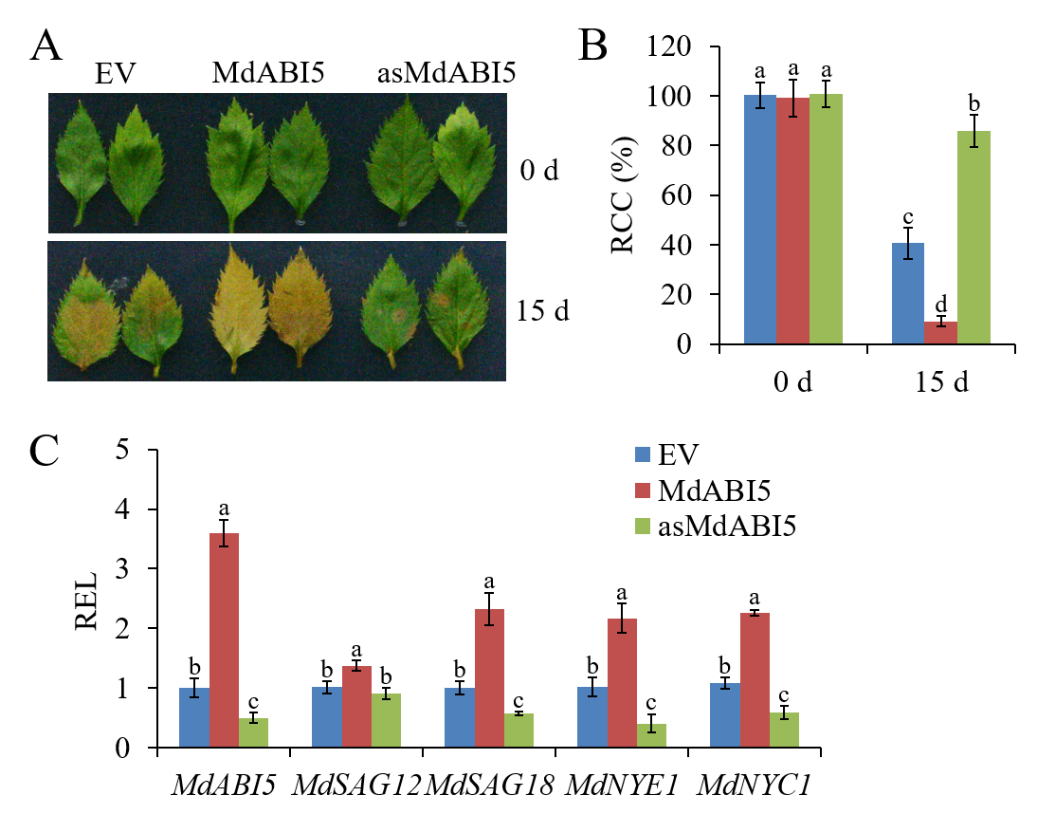


**Supplemental Figure 1. MdABI5 promotes leaf senescence in apple.**

(A) The senescence phenotype of *MdABI5* transient transgenic apple leaves before and after 15 d of dark treatment. Each treatment was performed in triplicate and each replicate comprised 8–10 apple leaves. Representative pictures are shown. EV, empty vector; MdABI5, *MdABI5*-overexpressing apple leaves; asMdABI5, apple leaves with suppression of *MdABI5*. (B) Relative chlorophyll content (RCC) in the apple leaves shown in A. The value for EV before dark treatment was set to 100% and used as the reference. (C) qRT-PCR analysis of the relative expression levels (REL) of *MdABI5*, *MdSAG12*, *MdSAG18*, *MdNYE1*, and *MdNYC1* in transient transgenic apple leaves. The value for EV was set to 1 and used as the reference. Three biological replicates were carried out with three technical repeats. Error bars denote standard deviations. Different lowercase letters indicate significant difference at *P* < 0.05 based on one-way ANOVA test.


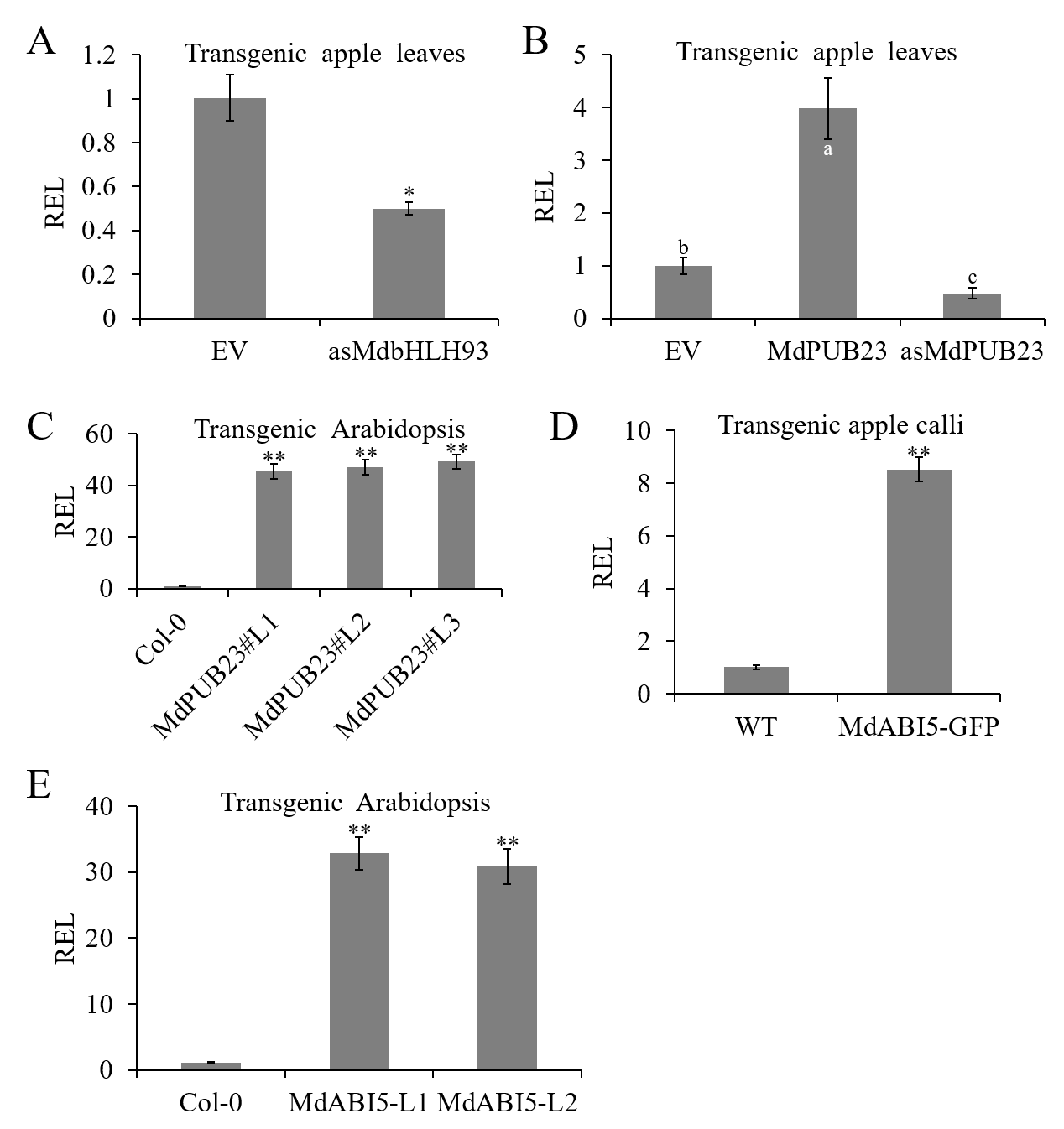


**Supplemental Figure 2. Identification of the transgenic plant materials.**

(A) qRT-PCR analysis of the relative expression level (REL) of *MdbHLH93* in transient transgenic apple leaves. EV, empty vector; asMdbHLH93, apple leaves with suppression of *MdbHLH93*. (B) qRT-PCR analysis of the REL of *MdPUB23* in transient transgenic apple leaves. EV, empty vector; MdPUB23, *MdPUB23*-overexpressing apple leaves; asMdPUB23, apple leaves with suppression of *MdPUB23*. (C) qRT-PCR analysis of the REL of *MdPUB23* in transgenic *Arabidopsis* seedlings. Col-0, wild type; MdPUB23#1, #2, and #3, *MdPUB23*-overexpressing *Arabidopsis* seedlings. (D) qRT-PCR analysis of the REL of *MdABI5* in transgenic apple calli. WT, wild type; MdABI5-GFP, *MdABI5*-overexpressing apple calli. (E) qRT-PCR analysis of the REL of *MdABI5* in transgenic *Arabidopsis* seedlings. MdABI5-L1 and L2, *MdABI5*-overexpressing *Arabidopsis* seedlings. The value for EV, WT, or Col-0 was set to 1 and used as the reference. Three biological replicates were carried out with three technical repeats. Error bars denote standard deviations. Different lowercase letters indicate significant difference at *P* < 0.05 based on one-way ANOVA test. Asterisks indicate statistical significance based on *t*-test. *, *P* < 0.05; **, *P* < 0.01.


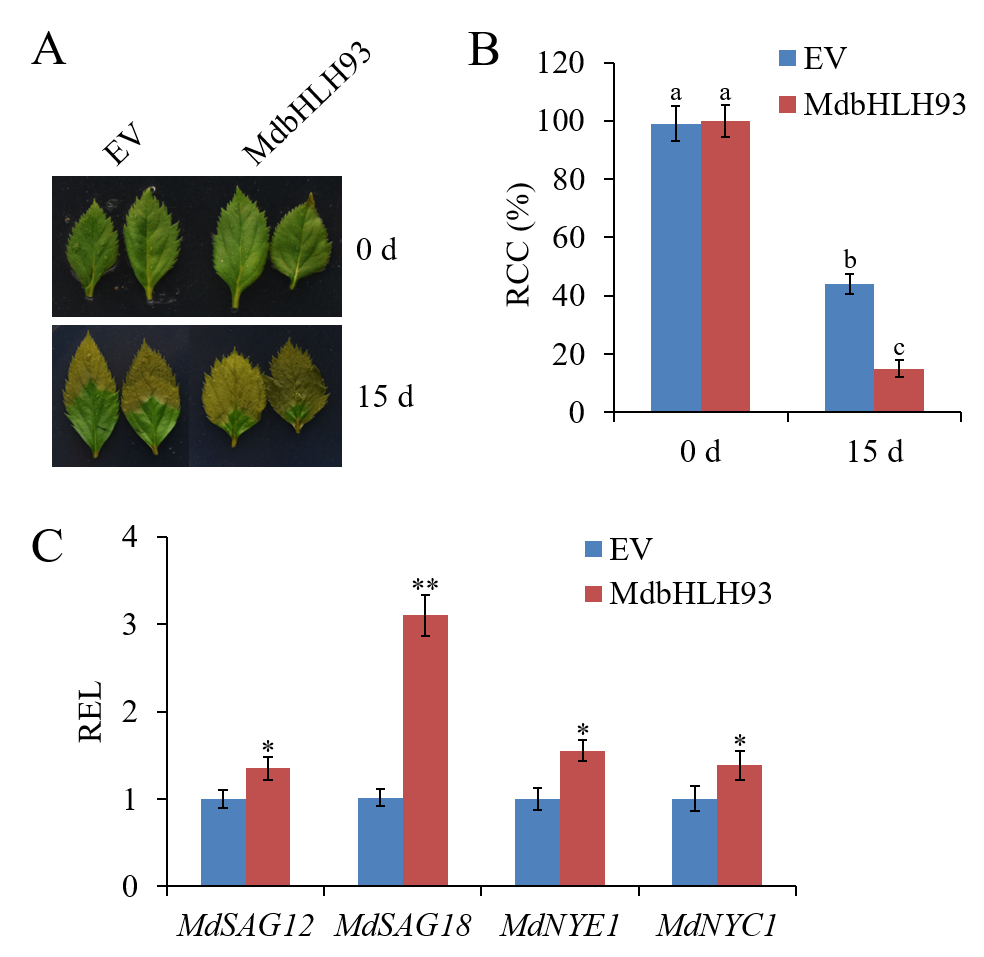


**Supplemental Figure 3.** **MdbHLH93 accelerates leaf senescence in apple.**

(A) The senescence phenotype of *MdbHLH93* transient transgenic apple leaves before and after 15 d of dark treatment. Each treatment was performed in triplicate and each replicate comprised 8–10 apple leaves. Representative pictures are shown. EV, empty vector; MdbHLH93, *MdbHLH93*-overexpressing apple leaves. (B) Relative chlorophyll content (RCC) in the apple leaves shown in A. The value for EV before dark treatment was set to 100% and used as the reference. (C) qRT-PCR analysis of the relative expression levels (REL) of *MdSAG12*, *MdSAG18*, *MdNYE1*, and *MdNYC1* in transient transgenic apple leaves. The value for EV was set to 1 and used as the reference. Three biological replicates were carried out with three technical repeats. Error bars denote standard deviations. Different lowercase letters indicate significant difference at *P* < 0.05 based on one-way ANOVA test. Asterisks indicate statistical significance based on *t*-test. *, *P* < 0.05; **, *P* < 0.01.


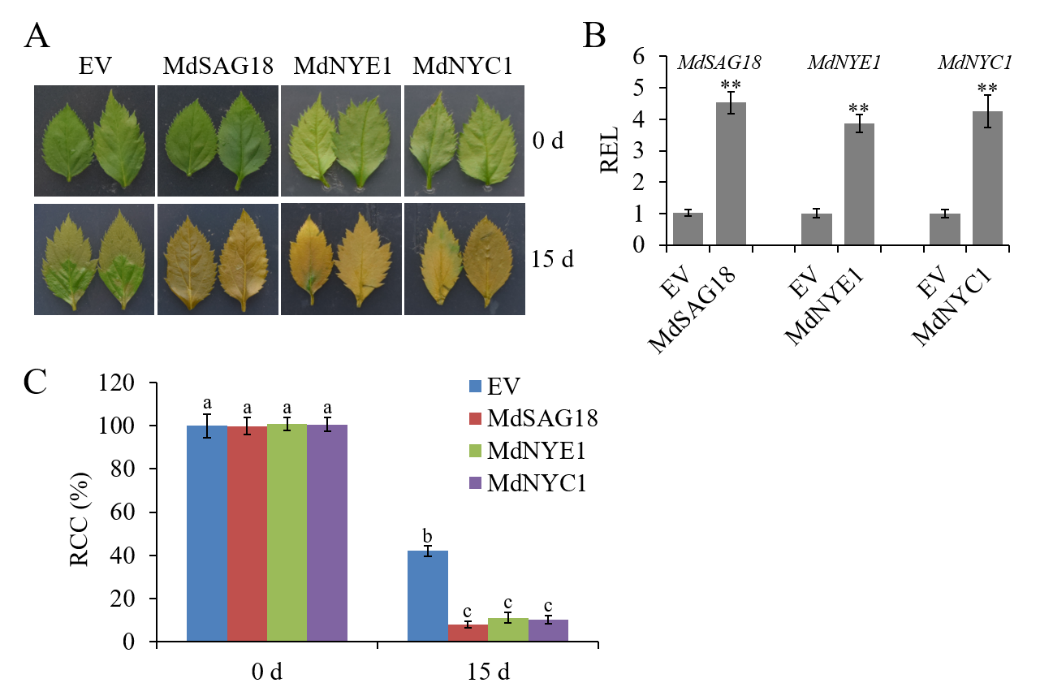


**Supplemental Figure 4. MdSAG18, MdNYE1, and MdNYC1 promote leaf senescence in apple.**

(A) The senescence phenotype of *MdSAG18*, *MdNYE1*, and *MdNYC1* transient transgenic apple leaves before and after 15 d of dark treatment. Each treatment was performed in triplicate and each replicate comprised 8–10 apple leaves. Representative pictures are shown. EV, empty vector; MdSAG18, *MdSAG18*-overexpressing apple leaves; MdNYE1, *MdNYE1*-overexpressing apple leaves; MdNYC1, *MdNYC1*-overexpressing apple leaves. (B) qRT-PCR analysis of the relative expression levels (REL) of *MdSAG18*, *MdNYE1*, and *MdNYC1* in transient transgenic apple leaves. The value for EV was set to 1 and used as the reference. Three biological replicates were carried out with three technical repeats. (C) Relative chlorophyll content (RCC) in the apple leaves shown in A. The value for EV before dark treatment was set to 100% and used as the reference. Error bars denote standard deviations. Different lowercase letters indicate significant difference at *P* < 0.05 based on one-way ANOVA test. Asterisks indicate statistical significance based on *t*-test. **, *P* < 0.01.


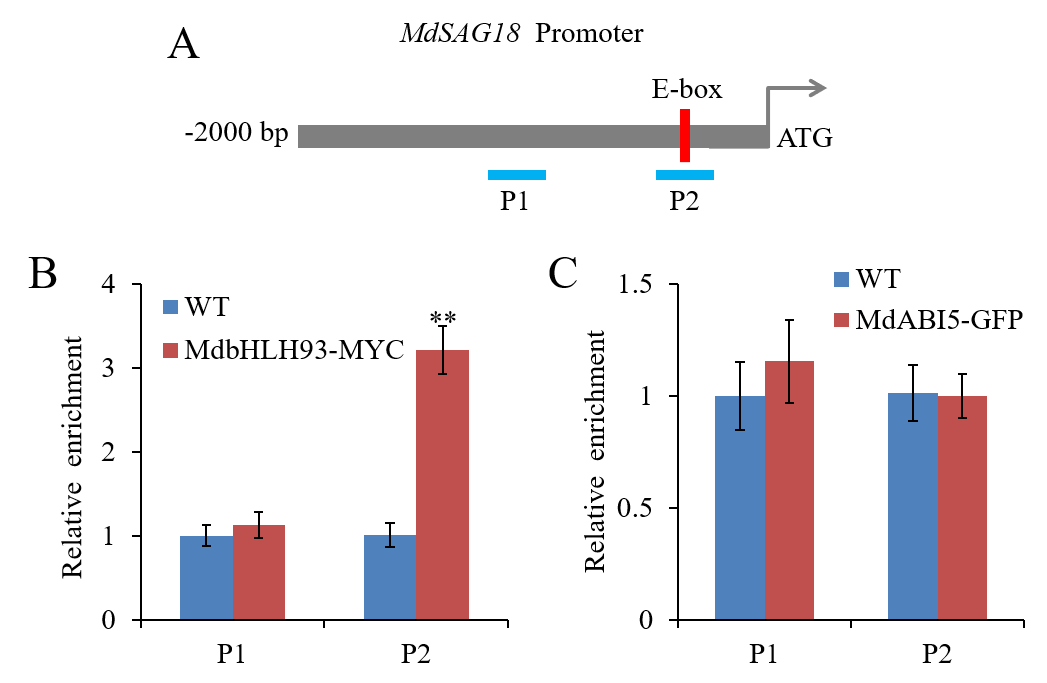


**Supplemental Figure 5.** **MdABI5 does not bind to the *MdSAG18* promoter.**

(A) Schematic diagram of the *MdSAG18* promoter. P1-P2 represent DNA fragments used for the ChIP-PCR assay. (B) ChIP-PCR assay showing the *in vivo* binding of MdbHLH93 to the *MdSAG18* promoter fragments. The value for WT was set to 1. WT, wild type; MdbHLH93-MYC, *MdbHLH93*-overexpressing apple calli. (C) ChIP-PCR assay showing that MdABI5 did not bind to the *MdSAG18* promoter. WT, wild type; MdABI5-GFP, *MdABI5*-overexpressing apple calli. Error bars denote standard deviations. Asterisks indicate statistical significance based on *t*-test. **, *P* < 0.01.

**
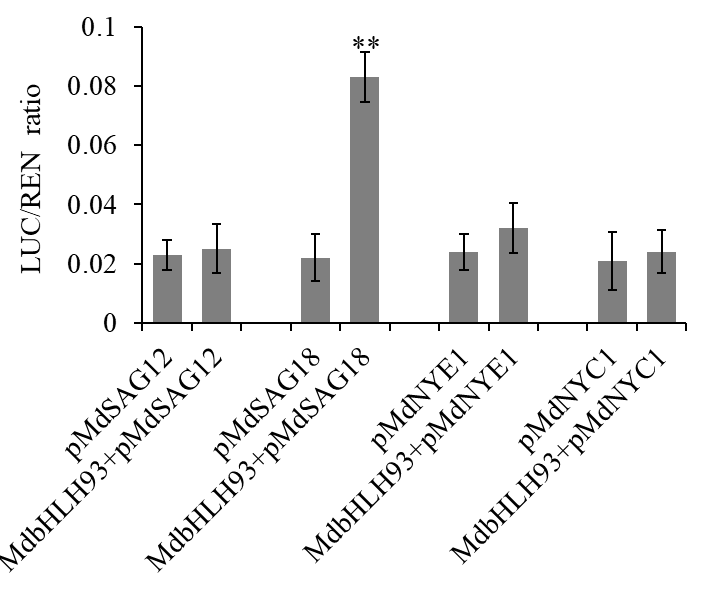
**

**Supplemental Figure 6.** **MdbHLH93 specifically activates the expression of the *MdSAG18* promoter.**

Dual luciferase assay. Error bars denote standard deviations. Asterisks indicate statistical significance based on *t*-test. **, *P* < 0.01.

**
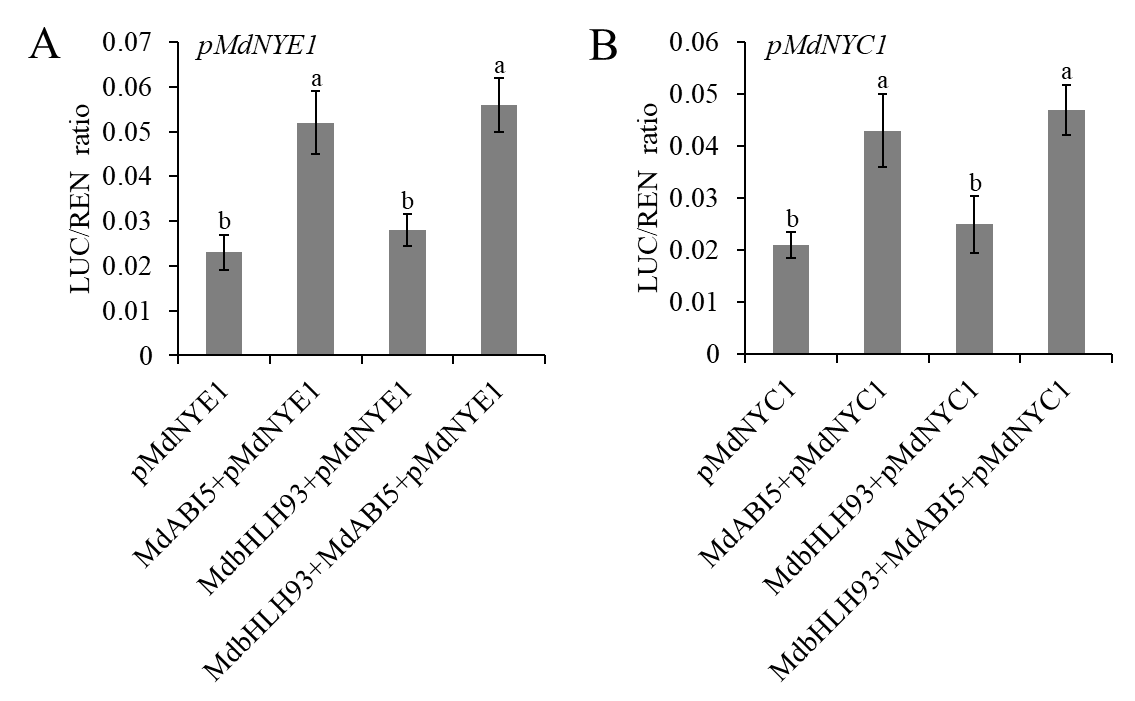
**

**Supplemental Figure 7.** **MdbHLH93 does not affect the transcriptional activation of MdABI5 to the *MdNYE1* and *MdNYC1* promoter.**

(A) Dual luciferase assay showing the effect of MdbHLH93 on MdABI5 transcriptional activation of *MdNYE1*. (B) Dual luciferase assay showing the effect of MdbHLH93 on MdABI5 transcriptional activation of *MdNYC1*. Error bars denote standard deviations. Different lowercase letters indicate significant difference at *P* < 0.05 based on one-way ANOVA test.

**
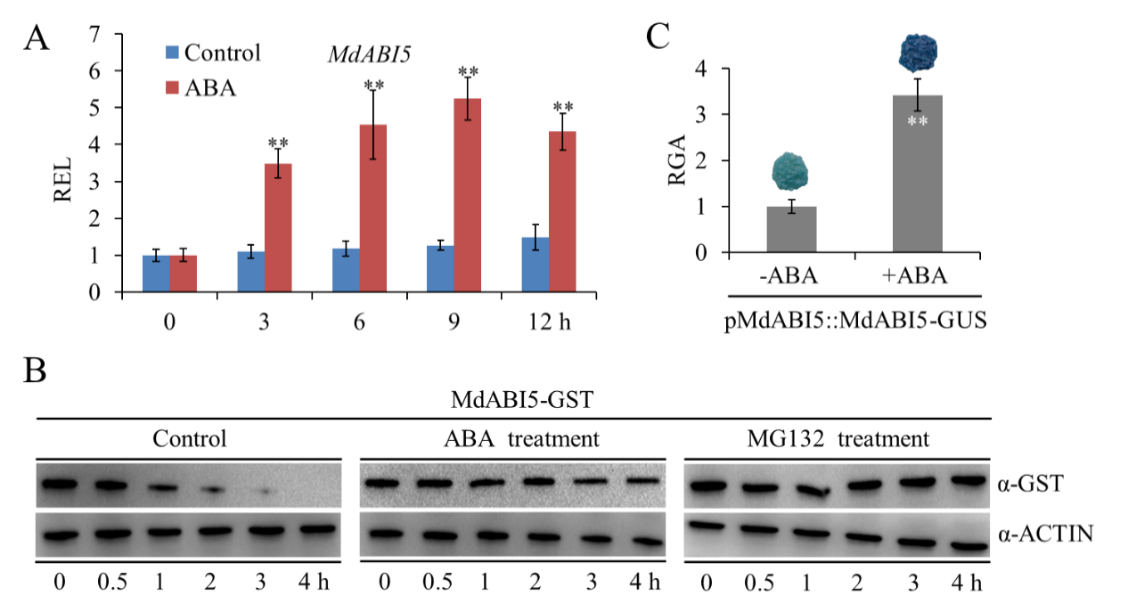
**

**Supplemental Figure 8. ABA treatment affects MdABI5 expression at the transcriptional and post-translational levels.**

(A) qRT-PCR analysis of the relative expression level (REL) of *MdABI5* after 50 μM ABA treatment for 12 h. The value for the control without any treatment was set to 1 and used as the reference. Three biological replicates were carried out with three technical repeats. (B) Western-blot analysis of the degradation of MdABI5-GST protein after treatment with 50 μM ABA or MG132 for 4 h. (C) GUS staining and relative GUS activity (RGA) of *MdABI5* transgenic apple calli in the absence and presence of ABA. The GUS activity for the apple calli without ABA treatment was set to 1 and used as the reference. Error bars denote standard deviations. Asterisks indicate statistical significance based on *t*-test. **, *P* < 0.01.


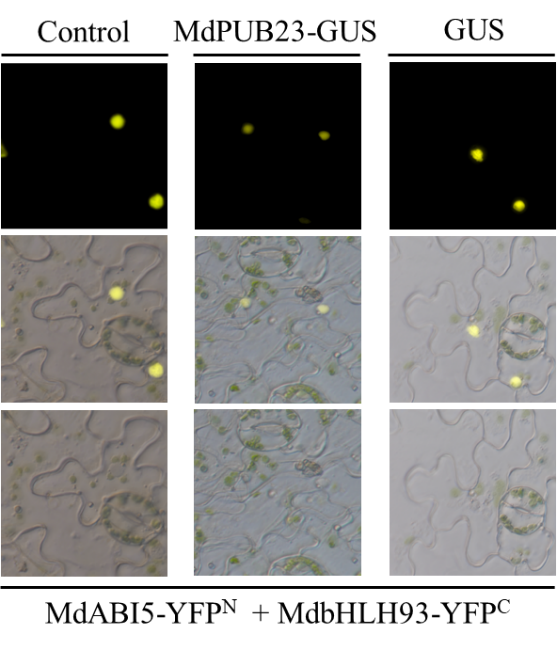


**Supplemental Figure 9. MdPUB23 interferes with the interaction between MdABI5 and MdbHLH93 in BiFC assays.**

**
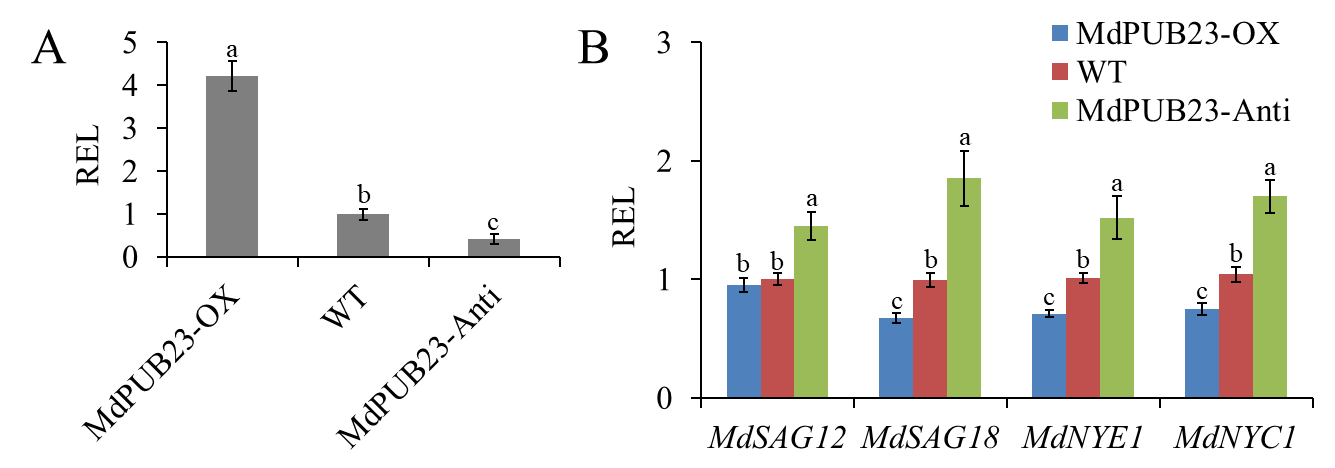
**

**Supplemental Figure 10. MdPUB23 negatively regulates the expression levels of senescence-associated genes in transgenic apple calli.**

(A) qRT-PCR analysis of the relative expression level (REL) of *MdPUB23* in transgenic apple calli. The value for WT was set to 1 and used as the reference. Three biological replicates were carried out with three technical repeats. WT, wild type; MdPUB23-OX, *MdPUB23*-overexpressing apple calli; MdPUB23-Anti, apple calli with suppression of *MdPUB23*. (B) qRT-PCR analysis of REL of *MdSAG12*, *MdSAG18*, *MdNYE1*, and *MdNYC1* in *MdPUB23* transgenic apple calli. Error bars denote standard deviations. Different lowercase letters indicate significant difference at *P* < 0.05 based on one-way ANOVA test.

**
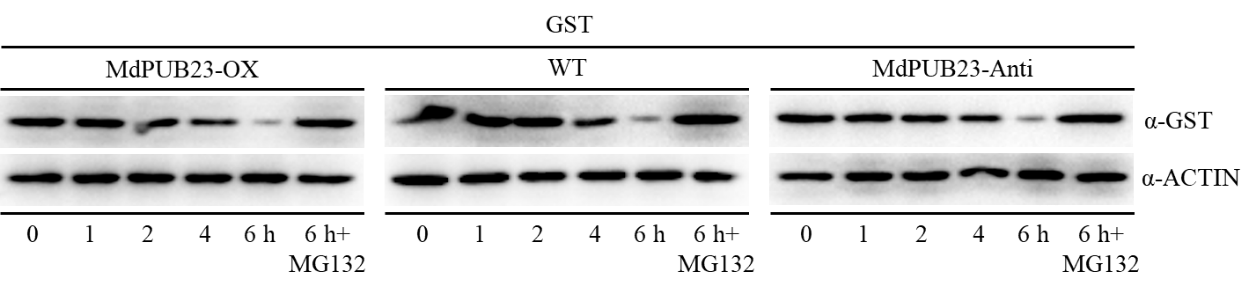
**

**Supplemental Figure 11. Protein degradation assays *in vitro*.**


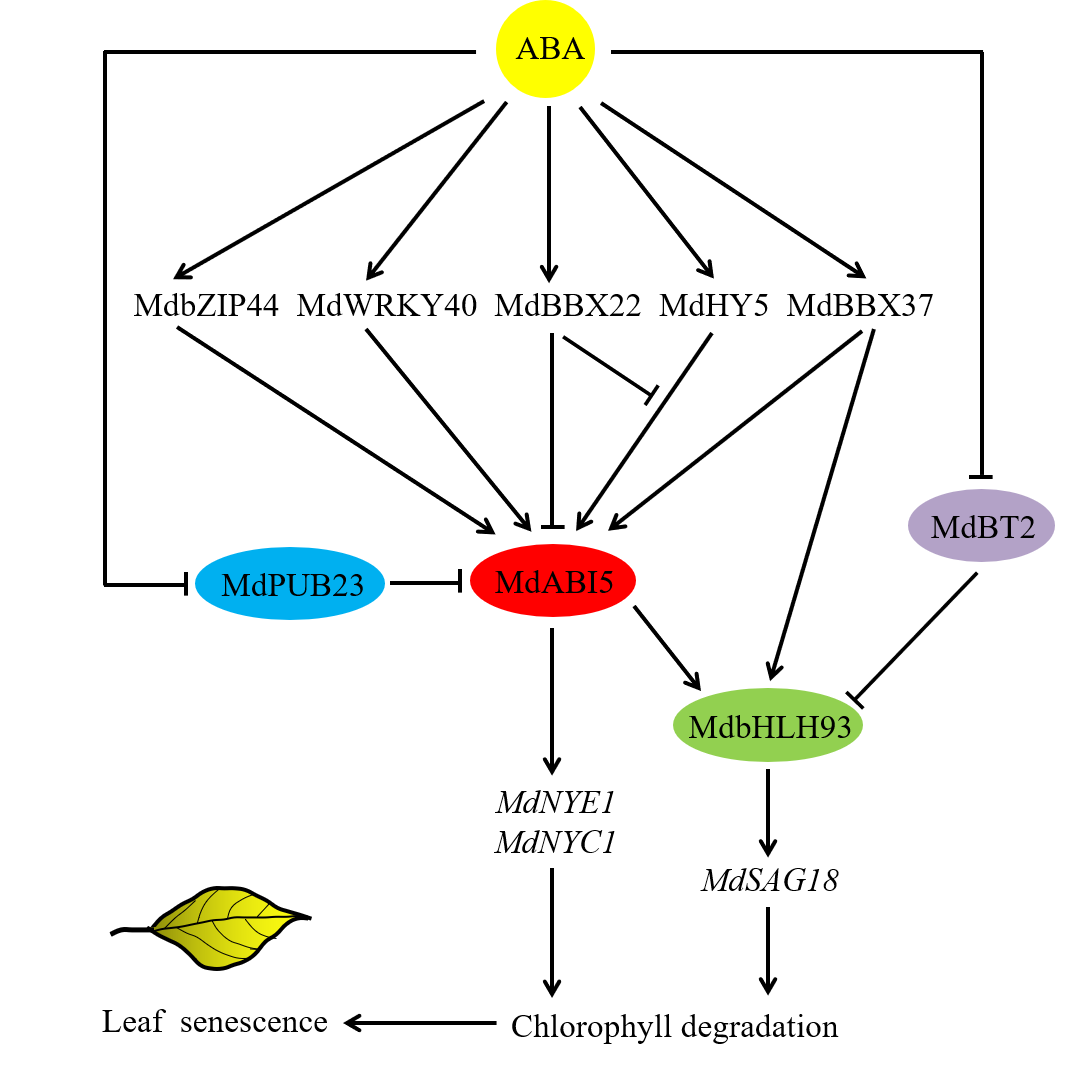


**Supplemental Figure 12. Model depicting the central role of MdABI5 in ABA-triggered leaf senescence in apple.**

**Supplemental Tables**

**Supplemental Table 1.** **Eight candidate partners that interact with MdABI5.**

The Y2H system was performed using MdABI5-pGBD to screen the MdABI5-interacting proteins. Eight proteins have been identified as candidate MdABI5-interacting proteins including MdbHLH93, MdPUB23, MdbHLH3, MdBBX22, MdbZIP44, MdWRKY40, MdZAT10, and MdTCP46.

**
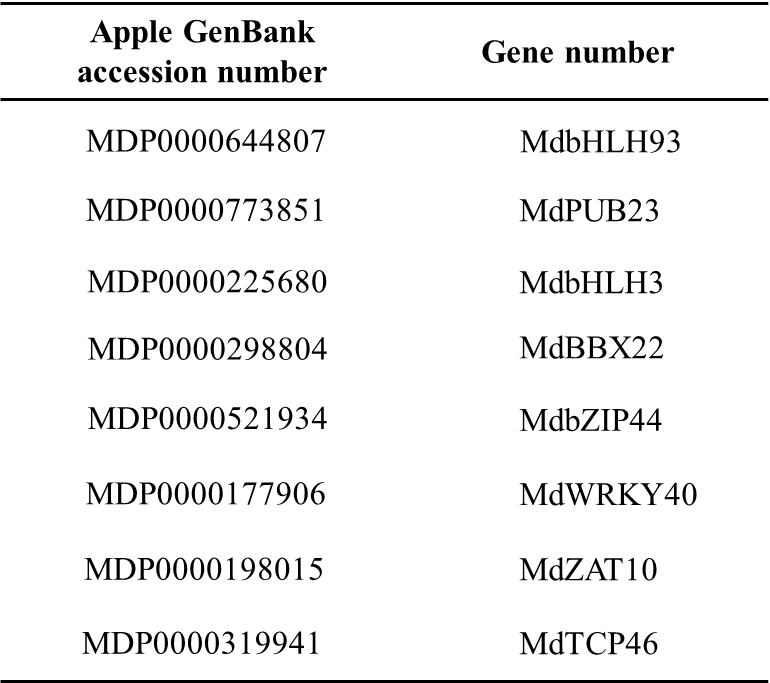
**

**Supplemental Table 2. The promoter sequence of *MdSAG18***

GCTCATTAGTCAATATAGCCGGAGTCACTACAATTACCTATACAACACTACACTGCACATAAGTCGGAACCACTAAAAGGGTCTGTACGACAAGACTAGGTGTAATATAATTATGCTCAATACTACGCTTTCATGATAGCTGGGCGATAAATCGCTAGTCACCTACGAGTCAGAACCACCTAAATTGGATCCAATGTGAGCATATGGTGTGGGAGGTGACATTATAAACATGCATGTGTCATATCTCTGGCTAAATCACAATCACCCTAGGTACATGTTTATGAGCTCAATGTTTCTCAATCACAACTTTAATCACATTCACAGTTCATAACTCACCTGGGCTTACCTGAGCGTCCACAGCACCACAATTATATAATATGCATCATATACCAATTCATATACAGAATATAAATTTATATGCATGGCATTTCAAGGCATACTTTCATTTAAACGCATTTTCTGGGAAAATATCAAGTATATAAATATATACTGAAAACCAAAAGCCCACTCACTAGTATGTCGAAGGGTCGTAGCCCCCGAGTCACTCTTGCATGCGCTCGTCCTCGGGATAAGTCTCCCCTATATGCGAAATAACTATAAAAAGGTTAATTTAAAGCACATAACCAATACTAACTAATAACTTATCATACAATGCTCAAATGAGGTATTTGAATATACCAACGGGATCTACACAACCTCATGAACATYCCCATATTTTTAGAAAAATTTTCTGACCACCCATGTGCCGGCCAAGGCACGGCTAGACGTGCCGCCCACGCGCGGGCACGTGCCAGGCGCACTGACAGCGGCAGTTAACACCGTCAGGAATATTCCGTCCAAAAACAAGCATAGACTGTTAATTATTAACGGCGTCATCTAACGTCGTTAGCATATTCCGTCAAAACTGACGGAATATGCCCTCTTCTTCTCCGATGAGTCGCCAGACGCCTGTGACTTTGCCGATTTCTGGATTTTTWTWTYAAAATTTTATATCTTTTTCATTTCTTAACCAAATTCTATGAAACTTGACCCATTTTGAAGATCTCACTAGGACGAACAAAATCATACCTGAGTCGAGTCCTGAAACCACCGGAAACCCGCTGGAAAAAGCCTCGATATTCCAGTAAAACTTAAAACTCATCGTTCTCGATAATCCAACGTCCAAATTCTTCCAACGAACCACTCCGAGCCTCGTGAGGACCTCCTTAAGCTACCTATAAGCTTGAAATCCTAAAAAACACACGATTTTACGATTGCATGAATAGTGACATAAATCGGGTTAGGGTTTTCACGAGTTTTCGAAAAAGTTCTTACCTAAAAATGGTACCATTCAACTCGTATGAACCTCAGGAACACGATGGTGTCCTTTAAACCCTCGATCTGCAAACTTTCCACTAGTTTTGTTGTTAATCTGTACGGGAGAAAAGAGAGAGAGTGAGTCCGAGGGAGAGAGCACGGGAGAGAAGAGAGAGAAATGTACTTGTGTGTGTGTGGGGGGGGTCCACGTGGGTCACCAACCAACCAAAGAAAACATTTAAAGTCCTAAGTGTTCCAAAACTTAGGAAAAATCTTAGAATTTGTCCGAATAAAACTTCACCCACATAACCACACAAAACGTCCAAGGGTAAATAACTAATCTCACGCAGTCGAAATTATATATCTCGGGACGGGCTGTGACAACAATTGTTCCACGTTATAACAAATTCGAGGGTCAATACTAACAAATATTTAGTTTCAAGGACCAAAACTTTAATTGAGTTAAAATTTAGGAATTATTACCCAATTACCTTTTAATTATAAGAAAAATTAATGAAAAGAAATTGAAAACTAGTATAGGAGTGACATTTTAAAGTAAAAATATGTTTAGCGTTAAAAATGAACATGAATGTGTGTTAAAACTATCGTAATATACGGAGACGTACAGTGGACAAAAAAGAGCAAGGGAACCAAACACTGTAAATTTGGAGGGGACGATTGGAGAACCAGAGAAGAGGAGGAAG

**Note: boxed nucleotides indicate the MdbHLH93 binding site**

**Supplemental Table 3. Primers used for gene expression analysis and vector construction**

| Primer name | sequence (5’ to 3’) |
| --- | --- |
| MdABI5-F  MdABI5-R  asMdABI5-F  asMdABI5-R  MdABI5-N-F  MdABI5-N-R  MdABI5-C-F  MdABI5-C-R  MdbHLH93-F  MdbHLH93-R  asMdbHLH93-F  asMdbHLH93-R  MdbHLH93-N-F  MdbHLH93-N-R  MdbHLH93-C-F  MdbHLH93-C-R  MdSAG18-probe-F  MdSAG18-probe-R  MdPUB23-F  MdPUB23-R  asMdPUB23-F  asMdPUB23-R  MdPUB23-N-F  MdPUB23-N-R  MdPUB23-C-F  MdPUB23-C-R  MdABI5(qRT)-F  MdABI5(qRT)-R  MdbHLH93(qRT)-F  MdbHLH93(qRT)-R  MdPUB23(qRT)-F  MdPUB23(qRT)-R  MdSAG12(qRT)-F  MdSAG12(qRT)-R  MdSAG18(qRT)-F  MdSAG18(qRT)-R  MdNYE1(qRT)-F  MdNYE1(qRT)-R  MdNYC1(qRT)-F  MdNYC1(qRT)-R  pMdSAG18-P1-F  pMdSAG18-P1-R  pMdSAG18-P2-F  pMdSAG18-P2-R  pMdSAG12-F  pMdSAG12-R  pMdSAG18-F  pMdSAG18-R  pMdNYE1-F  pMdNYE1-R  pMdNYC1-F  pMdNYC1-R | ATGTGCTTCAATTTGATCGGG  CAAACCACAACTATGACTCCT  CACTGCCTGCGCCACTGTGTA  TTCCCGAACTACCCCTGCTTT  ATGTGCTTCAATTTGATCGGG  TGTGGCTGCCATTGAT  GTGGTGCCTCCTCAGCCTC  CAAACCACAACTATGACTCCT  ATGGAGTTGAATGAACATGGT GAAGCATCTTCCTCCATATCC  ATGCTAAGATCAATTGTGCCCAAG GAATCTCAAGGCCTAAGGCTTCCA  ATGGAGTTGAATGAACATGGT  CTCCACACACATTCCAATGT  AGAAATACCAGAGCTAATA  GAAGCATCTTCCTCCATATCC  GGGGGGGGTCCACGTGGGTCACCAAC  GTTGGTGACCCACGTGGACCCCCCCC  ATGGACCAAGAAATCGACGT AGATGGATAAGAAGAAACCA  AGCAGGGGCGGTTATGGTCTTG TGAAGAACTAAGCACAGCTTCG  ATGGACCAAGAAATCGACGT  GTAGCTCCCACGTTGCAT  GAGTCTCGAGCGTACGCTG  AGATGGATAAGAAGAAACCA  AGTCCTCTGCGTATGCTGCGAATG ATCATCCTTCTCTGCCTCCTC  AAGACCTCAGCCCCAAAAATTCAA  CTTTTGAATGATTCCAAAGGTCCG  AGCAGGGGCGGTTATGGTCTTG  TGAAGAACTAAGCACAGCTTCG  AGATGTCCCTGCAAACAGTGA TTAGTCCCATCCTCGCTCACGC  TGAAAGGATTGATGAGAGGAA ACAAGATAGCCATCAGAGGAAT  ACTTCATCTTCTGCAAAGAATTAC TGGTGGTGGGCACCGCCTGATGAC  ATCACTTTTAAAGGAGTGCAAGCG TGTCAAGTAATTGATGGCCTTTCC  ATTTCTTAACCAAATTC  TTCGTTGGAAGAATTTGGA  AGAGAGAGAGTGAGTCCG  TCCTAAGTTTTGGAACAC  ATTACCAGCAACAATGGATTG  TTTTGTCCTGTTTGACGTTTG  GCTCATTAGTCAATATAGCCGG  CTTCCTCCTCTTCTCTGGT  ATGCTAAATAATATCAGTAG  CAAAAAAATATGTTTATATA  TTTTGGAGGCNTCCAAGT  TGCAGATAAAATAACACAGTGA |
